# Effects of Collagenase Preconditioning on Partially Incised Rat Tendon Treated with Light-Emitting Diodes and Platelet-Rich Plasma

**DOI:** 10.3390/biomedicines13051214

**Published:** 2025-05-16

**Authors:** Jihad A. M. Alzyoud, Abd Al-Rahman Salem Al-Shudiefat, Heba A. Ali, Samya A. Omoush, Dalal A. O. Shuqair

**Affiliations:** 1 Department of Basic Dental Sciences, Faculty of Dentistry, The Hashemite University, Zarqa 13133, Jordan; heba_ali@hu.edu.jo; 2 Department of Medical Laboratory Sciences, Faculty of Applied Medical Sciences, The Hashemite University, Zarqa 13133, Jordan; abdsalem29@hu.edu.jo (A.A.-R.S.A.-S.); samyah@hu.edu.jo (S.A.O.); 3 Department of Allied Basic Medical Sciences, Faculty of Applied Medical Sciences, The Hashemite University, Zarqa 13133, Jordan; dalal@hu.edu.jo

**Keywords:** collagenase, tendon, phototherapy, light-emitting diodes, platelet-rich plasma, tendinopathy

## Abstract

**Background:** Tendinopathy is a challenging condition associated with high treatment costs, prolonged dysfunction, and lower quality of life. Current treatment strategies aim to accelerate healing by modulating the healing phases. Phototherapy and growth factor-based modalities have shown promising outcomes in promoting tendon healing. A two-factor experimental design investigates the therapeutic efficacy of conditioning a partially tenotomized rat Achilles tendon model with low concentrations of collagenase, followed by platelet-rich plasma and/or light-emitting diode treatments. **Methods:** Forty-six adult male Wistar rats (284.8g ± 6.8) were randomly assigned to nine groups (G1 (n = 6), G2–G9; n = 5 per group) based on the treatment applied upon a partially incised rat’s hind-limb Achilles tendon model for three weeks. On day 21, blood samples were collected for hematological and biochemical analyses and tendon explants were harvested and subjected to histology. **Results:** Observational findings support the safety and validity of the model with insignificant weight gain. Hematological measures revealed no significant differences, except WBC, which was affected by phototherapy (*p* = 0.037). Blood biochemical measures of creatinine and AST levels were significantly affected by collagenase, while both treatments significantly influence CPK levels (*p* < 0.001). Histological scores revealed no significant main or interaction effect of both treatment modalities. Effect size estimates for biochemical variables were strong effects while hematological and histological variables demonstrated weak effects. **Conclusions:** Preconditioning a partially incised tendon with low collagenase and combined with PRP and/or LED therapy may offer therapeutic benefits by enhancing the remodeling phase of tendon repair. Study results validated the rat model, which could be a reliable model for future research to refine treatment as well as the investigational tools protocols.

## 1. Introduction

Achilles Tendinopathy encompasses all problems affecting Achilles tendons, including tendinitis (inflammation of the tendon) and tendinosis (degeneration of the tendon). Macromanifestations include pain, swelling, and dysfunction symptoms that result in high morbidity in the general population, especially in sports and work contexts, leading to an increase in treatment costs, prolonged dysfunction, and ultimately a lower quality of life [1,2]. Several internal and external factors were implicated in the development of tendinopathy such as low cellularity and vascularity and excessive overloads. These multi-factors coupled with a slow metabolic rate relative to other musculoskeletal system result in a slow healing process that might progress into tendon rupture. Pathological findings include microscopic tendon changes in disorganized collagen fibers, chondroplastic changes, inflammatory cell infiltrate, and cell death [3,4].

Treatment modalities have shifted from managing symptoms to potentiating the inflammatory, proliferative, and remodeling phases of tendon healing to regenerate the tendon to normal structure and function [5,6]. Treatments with regenerative abilities such as growth factors, phototherapeutic devices (photobiomodulation), and extracorporeal shock wave therapy were among the treatment modalities that demonstrated support for such effects. Light-emitting diode (LED) treatment is now applied for some medical conditions as a non-invasive modality in the skin (e.g., wound healing) and musculoskeletal disorders (e.g., tendinopathy) based on the principle of photobiomodulation using a biocompatible tissue-penetrating light as a non-invasive treatment that can enhance tissue healing and regeneration [7,8,9,10,11,12,13,14]. Platelet-rich plasma-based treatments for tendinopathies are based on the fact that PRP is a source of growth factors important in the tendon healing process, including insulin-like growth factor (IGF), transforming growth factor beta (TGF-B), and platelet-derived growth factor (PDGF), which modulate physiological processes such as cell proliferation, differentiation, and migration; collagen and proteoglycan synthesis; and angiogenesis [15,16,17]. Although these treatments showed encouraging results, prolonged treatment periods were needed, especially in chronic tendinopathies with ruptures [18,19].

In summary, outcomes of current tendinopathy treatments were slow and expensive and no methods were applied to improve the site of injury before the application of treatments. Novel methods in wound healing aimed at accelerating the healing phases, especially the remodeling phase, included the removal of injured extracellular matrix, especially the disorganized collagen fibers, to deposit new matrix components [19,20]. Collagenases, a subgroup of the matrix metalloproteinases (MMPs) that degrade collagens, have been used in cell cultures, as well as inducers in inflammatory models such as tendinitis and pancreatitis [21]. Collagenases of bacterial origin have recently been used as a treatment modality based on their ability to break down extra collagen or remove abnormal deposited collagen and promote wound healing [22]. For example, in wound healing and burns, collagenases were used therapeutically to selectively remove disorganized or necrotic tissue and enhance cell activity, thereby supporting tissue regeneration without inducing further injury [23,24]. The medicinal effects of (germ-free) maggots (i.e., the larva of the green bottle fly Lucilia sericata) have been supported as a biotherapy in the debridement of open wounds that resist treatment, based on their secretion of matrix proteinases [24,25].

The primary aim of this in vivo animal study is to assess the effect of conditioning a partially tenotomized rat Achilles tendon with low concentrations of collagenase, immediately before treatment application, on the healing process. Secondary aims include the assessment of the effect of LED and PRP treatments, applied as single or combined treatments, on the healing process of partially tenotomized rat tendons that were preconditioned with collagenase, comparing results across different treatment groups (LED-treated, PRP-treated, LED + PRP-treated) with and without collagenase preconditioning. Histomorphological, blood biochemistry, and hematological changes were the assessment methods used to accomplish these aims.

Therefore, it is hypothesized that the application of a low concentration dose of collagenase application at the site of a partially incised wound prior to treatment with LED and/or PRP will facilitate in the enzymatic debridement of disorganized abnormal collagen fibers at the site of the cut and, thereby, might help in accelerating the healing phases, especially the remodeling phase, by enhancing the matrix collagen fibers’ regeneration. To test this, an in vivo animal model was established to be used to investigate the application of the various treatment modalities combinations of low concentration of collagenase, LED, and PRP upon a partially incised rat Achilles tendon and measure their therapeutic efficacy on hematological, biochemistry, and histological features after a 21-day study period. To our knowledge, this is the first in vivo study to investigate the use of low-dose collagenase as a therapeutic agent in the treatment of tendon injury. This is described as a pilot study that employs a two-factor experimental design.

## 2. Materials and Methods

### 2.1. Study Design

This experimental in vivo study employed a two-factor experimental design to investigate the effects of collagenase preconditioning and different treatment modalities—light-emitting diodes (LED), platelet-rich plasma (PRP), and their combination—on the healing of partially incised rats Achilles tendon model. The in vivo animal model for partially incised rat Achilles tendon was approved by the ethical committee (Institutional Review Board) of the Hashemite University (reference number 3/3/2016/2017). Forty-six adult healthy male Wistar rats aged three months and weighing 284.8 g ± 6.8 were purchased from Jordan University of Science and Technology at Irbid, Jordan. They were housed in separate cages, were fed ad libitum under similar conditions at the animal house, and were acclimatized for three days. All rats were weighed at the start and the end of the experiment in the animal house at the Department of Biology and Biotechnology in the Faculty of Science at the Hashemite University. Rats with impaired mobility, food refusal, and sick signs were excluded. Sample size calculations were performed according to a formula presented by Arifin and Zahiruddin in 2017 that showed that 5 rats per group point per each group would be sufficient to have an 80% power and a 5% error [26]. Rats were randomly assigned using a computer-based random order generator to one of the following nine groups: In Group 1, six rats were left intact without any interventions and labeled as a normal group (G1). The remaining 40 rats underwent partial tenotomy of their Achilles tendon (right or left) and were randomly divided into eight groups based on the applied treatment (Table 1).

### 2.2. Surgical Procedure and Interventions

All surgical procedures were performed according to the ethical guidelines for using rats (Institutional Review Board of the Hashemite University, Jordan). Rats were anesthetized via intraperitoneal injection of a Ketamine (80 mg/kg) and Xylazine (10 mg/kg) mixture purchased from a local medical supply. The skin on the posterior surface of the rat’s hind limb was shaved, and a longitudinal incision was made to expose the Achilles tendon. A unilateral partial transverse section (i.e., perpendicular to the collagen fibers) of the Achilles tendon was made using a scalpel blade, which was approximately 3 mm in depth and 5 mm from the tendon’s insertion [27]. Before skin suturing, partially transected tendons were conditioned through direct contact application of a collagenase solution (1 u/µL DMEM; clostridium histolyticum, Sigma Aldrich, St. Louis, MO, USA) at the cutting ends for 1 min using a cotton pellet dipped in 1 mL of the collagenase conditioning solution. The low concentration of collagenase was used to allow the dissolving of collagen fibers at the injury interface without provoking an inflammatory response [28]. Subsequently, the area was washed with copious fresh phosphate-buffered saline (PBS) with EDTA to deactivate the collagenase. The collagenase conditioning medium was applied to groups G3, G4, G5, and G6. Collagenase was prepared from clostridium histolyticum lyophilized powder dissolved in plain fresh serum-free DMEM to produce a working 1 u/µL DMEM solution. The prepared solution was filter-sterilized (0.22 µL) before use. After surgery on the rats, the skin was sutured with a 3/0 surgical suture, disinfected with iodine, and rats were left free to move in singly housed cages. No medications were used after surgical procedures to prevent interventions of the drugs with the treatments.

The LED device (Photizo^®^, Photon Therapy Systems LTD, 2012, Pretoria, South Africa) standard probe (λ = 630 nm, energy output = 150 mW, and spot size = 1.2 cm^2^) was applied in direct contact with the site of injury in the Achilles tendon at an energy density of 4 J/cm^2^, delivered every other day until the end of the experiment. Placebo sham treatments were performed using a normal non-LED light bulb. Fresh homologous platelet-enriched plasma was prepared according to standard methods [10]. Briefly, 10 mL blood samples were centrifuged at 2500 rpm at 22 °C for 6 min, following which the supernatant and buffy coat were taken and re-centrifuged at 4200 rpm at 22 °C for 7 min. Two-thirds of the supernatant were discarded, and the platelet pellet was re-suspended in the remaining third of the supernatant. The prepared PRP was administered in the form of subcutaneous (0.2 mL) injections at the incision site once a week, starting immediately after surgery (i.e., day one). Placebo treatments were performed using 0.2 mL of saline. In combined treatments, LED treatments were performed before PRP injections. All interventions were extended to the end of the experiment (i.e., 21 days). On the 21st day of the experiment, the animals were anesthetized, and the needle cardiac puncture method was used to collect blood samples (5 mL) in plain and EDTA tubes for hematology and biochemistry, respectively. Animals were euthanized according to the ethical principles of animal welfare, and Achilles tendons were excised for routine histological processing [29].

### 2.3. Observational, Blood Biochemistry and Hematology

Observational notes were recorded for eating and walking ability during the activity. Blood samples collected in tubes with EDTA as an anticoagulant were used to determine hemoglobin (Hb) concentration, packed cell volume (PCV), and white blood cell count (WBC). Blood samples collected in plain tubes were left standing for 15 min to confirm clotting, then centrifuged immediately at 4000 rpm for 20 min. The harvested serum was used to measure creatinine, aspartate aminotransferase (AST), alanine aminotransferase (ALT), creatine phosphokinase (CPK), and C-reactive protein (CRP) levels. These markers were used to evaluate inflammatory and metabolic responses and potential changes in vital organ functions.

### 2.4. Histological Assessment

The collected tendon explants (five per group) were washed with PBS, blotted dry using filter paper and immediately fixed by immersion in fresh 10% (*v*/*v*) neutral buffered formalin solution (NBFS) for 48 h at room temperature. Fixed tendon samples were embedded in paraffin wax in the desired orientation using an automated embedding device (Microm, GmbH, Walldorf, Germany) located at the Department of Medical Laboratory Sciences of the Hashemite University. Paraffin-embedded blocks were used to produce sections with a thickness of 7 µm using a microtome (Microm, GmbH, Walldorf, Germany) and were picked up on slides, warmed on a hot plate at 60 °C for 5 min, immersed in xylene twice for 5 min each, then rehydrated in serial ethanol solutions and washed in double-distilled water, ready for staining with Hematoxylin and Eosin (H&E) and Sirius red picrate special stains [30]. Sirius red picrate stain (acidic dye) was used to stain collagen fibers based on the birefringent properties of stained sections under a polarized light microscope, which enabled the differentiation between collagen types I and III [30,31,32]. For this purpose, a Sirius red picrate stain kit (Bio-Optica, Milan, Italy) was used to stain paraffin-embedded sections, following the manufacturer’s protocol. Briefly, slides were deparaffinized in two changes in xylene, rehydrated using sequential ethanol grades, and subsequently incubated in Sirius red picrate stain solutions. Slides were then placed in 70% ethanol for 5 min, followed by two changes in absolute ethanol, and cleared in two changes in xylene before mounting in DPX.

### 2.5. Imaging and Scoring

A light microscope (OPTIKA, Milan, Italy) and polarized microscope (OPTIKA, Italy) equipped with a digital camera (OPTIKA, Milan, Italy) and a computer were used to examine and take images for the H&E- and Sirius red picrate-stained slides, respectively. Images were captured at a magnification power of 100× and 400× for H&E slides and 100× for Sirius red picture slides using the Optika PROVIEW software (version 4.8; Ponteranica, Italy). Three slides were selected for each tendon explant and two images were taken per slide, labeled, and saved. Images were analyzed for the effects of LED and/or PRP treatments on the tendon matrix based on previous scoring systems [33]. Two histological features were examined and scored; first, the extracellular matrix organization, for which the wavy appearance of collagen fibers was scored based on a four-point system—ranging from 4: coarse crimp to 1: complete disarray (woven)—using the polarized Sirius red picrate images. Second, the cellularity of tenocytes was examined, according to their cell counts and shape, based on a four-point scoring system—ranging from 4 (indicating normal count or flattened cells) to 1 (indicating high cellularity or round cells)—using the light H&E images (Table 2). Two trained and blinded operators were recruited to score the digital images for one phase, and the data obtained were presented as means and standard deviations of the overall average score of each group were calculated as an average of the three histology features.

### 2.6. Statistical Analysis

Statistical analyses were performed using IBM SPSS Statistics software package, version 27 and GraphPad Prism software, version 10. Data cleaning was conducted to identify and address outliers and missing values, all reading. The collected data were summarized using tables and figures and presented as mean ± standard deviation (SD). Normality of data were tested using the Shapiro–Wilk test and homogeneity of variances were assessed using the Levene’s tests. Since the experimental setup is a two-way factorial design that included a two independent variables, collagenase preconditioning (Yes/No) and phototherapy treatment (PRP, LED, PRP + LED, normally distributed data were analyzed using a normal two-way factorial ANOVA model to assess the main effects of collagenase preconditioning and phototherapy treatment, as well as their interaction, on each measured outcome (e.g., biochemical, hematological, and histological scores). Non-parametric Aligned Rank Transform (ART) two-way ANOVA tests were used for non-normally distributed measures who failed the normality following transformation. The effect size point estimate was reported for each factor using the Partial Eta-Squared (partial η^2^) measure and its values were classified as small (~0.01), medium (~0.06), or large (~0.14). Coefficient of determination (R^2^) is another reported estimate of effect size but for the whole model. A *p*-value of <0.05 was considered statistically significant, and 95% confidence intervals were reported where appropriate. A sample size of five rats per group was determined to provide 80% power at a 5% significance level [26].

## 3. Results

### 3.1. Observational Changes, Blood Biochemistry, and Hematology Analysis

**Figure 1:**
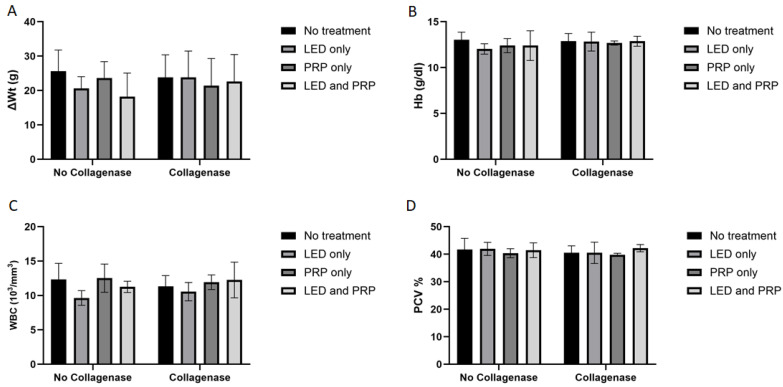
Summary of the weight changes and hematological data (mean score ± standard deviations) for all groups on day 21. (**A**) Weight change; (**B**) hemoglobin (HB); (**C**) white blood cell (WBC); (**D**) packed cell volume (PCV). Bar graphs created using GraphPad Prism software, version 10.

Decreased activity of eating and walking was observed for all the rats (except those in group G1) during the first two days following the surgery, while diet intakes remained normal for the duration of the experiment. The shaved hair at the site of injury grew normally, the site of the skin incision almost healed with no signs of infections, and rat walking activity was restored to almost normal at the end of the experiment in all groups (i.e., day 21). Results of the Shapiro–Wilk normality test and the Levene’s homogeneity of variances test for histological, biochemical, and hematological outcomes are presented in Supplementary File S1. Measured data for body weight change (ΔWt) indicated that all groups of rats had gained some weight by the end of the experiment (Figure 1A). Statistically, results of the Aligned Ranks Transformation Two-Way ANOVA test of weight changes showed no significant differences in the main effect of phototherapy (F (3, 38) = 0.381, *p* = 0.767, partial η^2^ = 0.029), or collagenase (F (1, 38) = 0.538, *p* = 0.468, partial η^2^ = 0.014), or their interaction (F (3, 38) = 0.707, *p* = 0.554, partial η^2^ = 0.053). Hematology data for hemoglobin (HB) (Figure 1B) tested using the normal Two-way ANOVA test have also showed no significant differences in the main effect of phytotherapy (F (3, 38) = 0.499, *p* = 0.685, η^2^ = 0.038), or collagenase (F (1, 38) = 2.086, *p* = 0.157, η^2^ = 0.052), or their interaction (F (3, 38) = 0.380, *p* = 0.768, η^2^ = 0.029).

**Figure 2:**
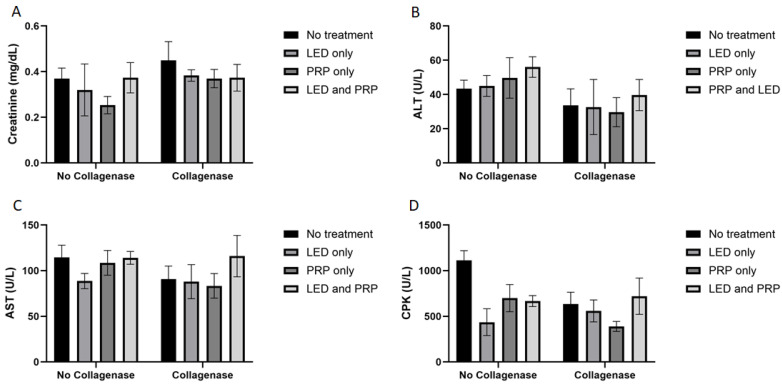
Summary of the blood biochemistry data (mean score ± standard deviations) for all groups on day 21. (**A**) Creatinine; (**B**) alanine aminotransferase (ALT); (**C**) aspartate aminotransferase (AST); (**D**) creatine phosphokinase (CPK). Bar graphs created using GraphPad Prism software, version 10.

White blood cell count (WBC) data (Figure 1C) have shown a significant effect of phototherapy (F (3, 38) = 3.120, *p* = 0.037, η^2^ = 0.198) while collagenase (F (1, 38) = 0.206, *p* = 0.653, η^2^ = 0.005) and the interaction between collagenase and phototherapy (F (3, 38) = 0.707, *p* = 0.554, η^2^ = 0.053) were not significant. Results for packed cell volume (PCV) (Figure 1D) showed no significant effects of phototherapy (F (3, 38) = 0.705, *p* = 0.555, η^2^ = 0.053), collagenase (F (1, 38) = 0.998, *p* = 0.324, η^2^ = 0.026), or their interaction (F (3, 38) = 0.051, *p* = 0.985, η^2^ = 0.004). Blood biochemistry data (Figure 2A) tested using the normal Two-way ANOVA test have shown that creatinine at the end of the experiment revealed that collagenase significantly affects creatinine levels (F (1, 22) = 7.076, *p* = 0.014, η^2^ = 0.243), while phototherapy showed no significant effects (F (3, 22) = 1.396, *p* = 0.270, η^2^ = 0.160). Phototherapy and collagenase interaction also showed no significant effect (F (3, 22) = 1.024, *p* = 0.401, η^2^ = 0.123). Alanine Aminotransferase (ALT) data (Figure 2B) showed significant main effect of phototherapy (F (3, 22) = 2.777, *p* = 0.065, η^2^ = 0.275) and collagenase (F (1, 22) = 4.142, *p* = 0.054, η^2^ = 0.158), while their interaction was not significant (F (3, 22) = 1.651, *p* = 0.207, η^2^ = 0.184). Aspartate aminotransferase (AST) data (Figure 2C) showed a significant main effect of collagenase (F (1, 22) = 13.383, *p* = 0.001, η^2^ = 0.378), while no significant were seen for phototherapy (F (3, 22) = 1.143, *p* = 0.354, η^2^ = 0.135) and the interaction between phototherapy and collagenases (F (3, 22) = 0.308, *p* = 0.819, η^2^ = 0.040). Creatine phosphokinase (CPK) data (Figure 2D) showed significant main effect for phototherapy (F (3, 22) = 13.095, *p* < 0.001, η^2^ = 0.641), collagenase (F (1, 22) = 9.595, *p* = 0.005, η^2^ = 0.304), and their interaction (F (3, 22) = 9.134, *p* < 0.001, η^2^ = 0.555) (Supplementary Files S2 and S3).

### 3.2. Histological Findings

**Figure 3:**
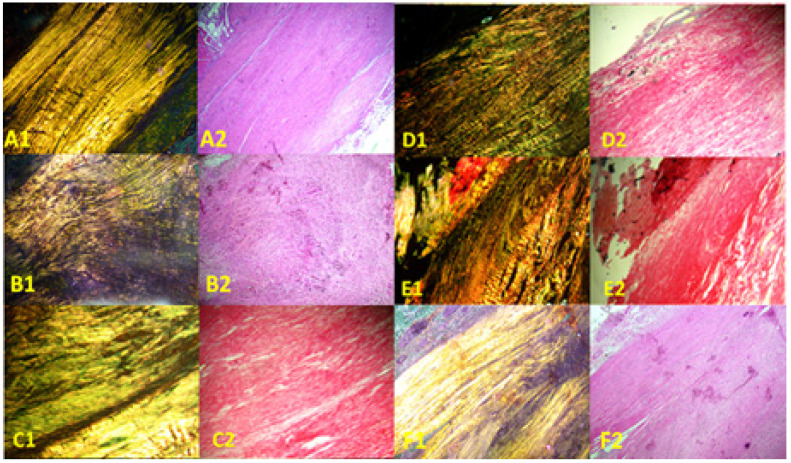
Photomicrographs of longitudinal sections of rat tendons stained with Sirius red picrate stain. Right column, non-polarized images; left column, polarized images. (**A1**,**A2**) Healthy rats (normal control group, G1), showing wavy parallel normal appearance; (**B1**,**B2**) partially injured rats (model group, G2) showing disorganized collagen fibers; (**C1**,**C2**) collagenase-conditioned rats (G3); (**D1**,**D2**) LED-treated rats with collagenase (G4); (**E1**,**E2**) PRP-treated rats with collagenase (G5); (**F1**,**F2**) LED and PRP-treated rats with collagenase (G6). Mag. 100×.

**Figure 4:**
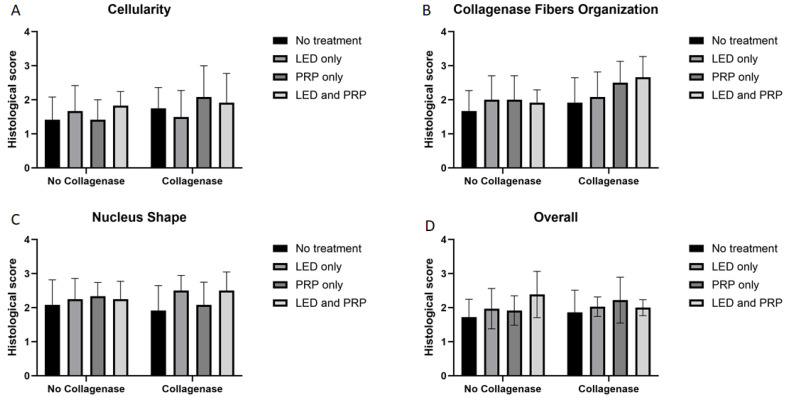
Summary of the histological scores (mean score ± standard deviations) for all groups on day 21. (**A**) Cellularity; (**B**) collagen fiber organization; (**C**) nucleus shape; (**D**) overall histological score. Bar graphs created using GraphPad Prism software, version 10.

Images of the normal control group (G1) showed normal histological features of parallel arranged collagen fibers and intact surfaces. In contrast, all other groups showed tissue-increased changes (to varying degrees) in collagen fiber arrangement (Figure 3). It was noted that healed tissues in all treated groups with/without collagenase conditioned (i.e., G3–G9) presented as incomplete healing of the incised part with granulation tissues (remodeled phase), while the adjacent areas to the incised part showed improved remodeled phase, with more flattened nuclei and better organization of collagen fibers with decreased cellularity (Figure 3 and Figure 4). Aligned Ranks Transformation Two-Way ANOVA tests for histological outcomes were performed and the main effects of phototherapy, collagenase, and their interaction were reported. Cellularity data (Figure 4A) showed no significant main effects for phototherapy (F (3, 46) = 0.776, *p* = 0.513, partial η^2^ = 0.048) and collagenase (F (1, 46) = 0.027, *p* = 0.871, partial η^2^ = 0.001), or their interaction (F (3, 46) = 1.069, *p* = 0.371, partial η^2^ = 0.065). Collagen fiber organization data (Figure 4B) showed no significant effect for phototherapy treatment (F (3, 46) = 0.211, *p* = 0.888, partial η^2^ = 0.014) or collagenase (F (1, 46) = 0.654, *p* = 0.423, partial η^2^ = 0.014), or their interaction (F (3, 46) = 2.266, *p* = 0.093, partial η^2^ = 0.129). Similarly, nucleus shape results (Figure 4C) showed that neither phototherapy F (3, 46) = 0.191, *p* = 0.902, partial η^2^ = 0.012) nor collagenase (F (1, 46) = 0.569, *p* = 0.454, partial η^2^ = 0.012), nor the interaction between phototherapy and collagenase (F (3, 46) = 2.207, *p* = 0.100, partial η^2^ = 0.126) had reached the significant level. Finally, the overall average score of the three histological features (Figure 4D) also showed no significant main effect of phototherapy (F (3, 46) = 0.183, *p* = 0.908, partial η^2^ = 0.012), or collagenase F (1, 46) = 0.055, *p* = 0.816, partial η^2^ = 0.001), and their interaction (F (3, 46) = 1.447, *p* = 0.241, partial η^2^ = 0.086) (Supplementary Files S2 and S3).

### 3.3. Effect Size η^2^ and R^2^ Interpretations

Results of the Partial Eta-Squared (partial η^2^) and the coefficient of determination (R^2^) are presented in Table 3 and Table 4, respectively. Results of the point estimate for effect size using the partial η^2^ and the R^2^ showed the variations among the measured outcomes. In hematological and histological outcomes, partial η^2^ ranged from small to medium effect for phototherapy, collagenase, and their interactions. These findings denoted a medium effect of the treatment. In biochemical outcomes the partial η^2^ effect range from medium to large for phototherapy, collagenase, and their interactions. These findings denoted a strong effect of the treatment. The R^2^ denoted how the dependent variable is explained by the independent variables as high values represent a good model fit. In hematological and biochemical markers, R^2^ values ranged from low to high denoting a moderate model fits and indicates more variability is explained by the model while histological outcomes showed low R^2^ values denoting a weak model fits indicates less variability is explained by the model.

## 4. Discussion

The healing of a partially transected tendon involves a cascade of three phases—an inflammatory phase, a proliferative phase, and a remodeling phase—with the eventual aim of restoring the injured tendon’s normal anatomy, histology, and physiology [34]. It starts with a short initial inflammatory response that recruits several types of cells and elicits a cell proliferation phase, which results in the synthesis of collagen fibers and ground substance, which marks the beginning of the long phase of matrix remodeling [35]. Therefore, signs of healing largely depend on matrix cellularity (i.e., cell count, shape, and viability) during the early phase and the degree of matrix organization (i.e., content and spatial arrangement of collagen fibers) during the late phase [36,37]. The factorial design of the experiment allows us to examine the main effect of collagenase preconditioning and phototherapy individually or synergistically upon the healing of a partially transected tendon of an in vivo rat model. The observational results of the conducted study showed that rats subjected to partial tenotomy conditioned with collagenase and treated with PRP and/or LED presented restored function and overlying skin at the end of the 21 days.

Histological results revealed no significant effect in the overall model neither of the dependent factors (i.e., phytotherapy or collagenase) nor their interactions. Notably, collagen fiber organization and nucleus shape *p* values support our observational findings, as collagen fibers organization and nucleus morphology scores in treated groups (G3–G9) did not differ from the normal control group (G1), suggesting improved healing relative to the untreated model (G2), although full restoration was not achieved. These observed results reflect the gradual restoration of cellular morphology during tendon remodeling phase, potentially driven by mechanotransduction signals that regulate tenocyte shape and collagen alignment in response to physiological loading. This is consistent with the observed recovery of rat mobility. Previous studies have shown that mechanical stimulation during tendon healing influences cytoskeletal organization and cell morphology, supporting the alignment of collagen fibers and restoration of flattened shaped nuclei with no significant main effects or interaction [38]. These findings merit further investigation. Moreover, the significant decrease in collagen organization, nucleus shape, and increased cellularity in G2 compared to G1 (Mann–Whitney U test, *p* value < 0.05) reinforces the validity of the in vivo partially tenotomized tendon rat model, showing distinct histopathological changes indicative of tendon damage and incomplete healing.

Hematology and biochemistry data are important indicators of the general health of animals, which maintained the validity of the model when compared to normal observational data and provide insight on the healing effect of treatment modalities [39]. Hematological results revealed no significant effect in the overall model neither of the dependent factors (i.e., phytotherapy or collagenase) nor their interactions for HB and PCV. Phototherapy alone has significant effect on WBC. Biochemical results showed significant effect in the overall model for ALT and CPK only. AST outcomes showed a significant effect of collagenase alone, albeit the overall model was not significant. CPK data showed a significant effect of individual dependent factors (i.e., phytotherapy or collagenase) and their interactions. Weight changes revealed no significant main effects or interaction for the collagenase and phototherapy modalities.

Several studies investigating phototherapy and PRP have supported their positive outcomes [33,40,41,42,43]. An in vitro study has reported increased cell viability when performing LED (625 nm and 850 nm) and PRP treatment on sheep tenocytes [10]. Another in vivo animal study showed improved histological features and mechanical properties when performing 650 nm and 1.8 J/cm^2^ laser therapy and PRP [33]. An animal in vivo study has reported that PRP and laser therapy improved the remodeling of type I and III collagen fibers, which are important structural components of the tendon matrix [41]. Our results are consistent with previous studies, with increased cellularity being a prominent feature reported in injury repair models, which is recognized as a hallmark of the proliferative phase of the healing response [33,40]. LED and PRP may have accelerated healing throughout the experimental period; however, it is apparent from the histological results that the healing response occurred in the remodeling phase (i.e., after 2 weeks). The chondroplastic changes represented by round nuclei were due to a decrease in the movement of the rats due to injury, which has been supported by findings in ruptured tendons where cells appeared to be more round [38]. The insignificant differences between the model group (G2) and the collagenase-conditioned group (G3) (Mann–Whitney U test, *p* value > 0.05) support the potential role of collagenase as a preparatory step before further regenerative treatments, consistent with previous reports on enzymatic debridement facilitating tissue repair [22,23].

The results of this study provide some evidence that combined treatment of phototherapy and collagenase preconditioning for 21 days might have the potential to enhance the healing of partially tenotomized Achilles tendons through the modulation of the healing process, including nucleus morphology and collagen fiber organization. The effect was detected after 21 days, thus spanning all phases of healing, which might explain the insignificant differences between the normal control group and the remaining treated groups. PRP produces healing effects through the release of different growth factors that are essential for cell metabolism [41,44,45,46,47], while LED photobiomodulation produces healing effects through chemical and thermal interactions with tissue chromophores that take place at certain wavelengths (e.g., 880 nm and 630 nm) [48,49]. The application of collagenases showed improved results, which supports the hypothesis regarding the role of collagenases in the removal of disorganized abnormal collagen fibers at the site of the cut, thus potentially accelerating the process of remodeling. Although collagenases may induce an inflammatory response, the small concentration used in this study is thought to improve the inflammatory phase, rather than deteriorate it.

Finally, it is important to note that this study was conducted as a pilot investigation, and the small sample size limits the statistical power and generalizability of the findings. While the findings provide preliminary insights, they should be interpreted with caution. Moreover, the absence of earlier histological and biochemical sampling points may have limited our understanding of dynamic matrix changes in the early inflammatory and proliferative phases. The study also relied primarily on histological analysis without assessing biomechanical properties of the tendon, which could further validate the observed remodeling effects. Future studies addressing these limitations with larger sample sizes, time-series sampling, and functional outcome measurements are warranted to build on the findings of this pilot investigation.

The factorial design allowed for efficient evaluation of interaction effects, the relatively small group sizes (n = 5) limited the statistical power, particularly for detecting interactions. Consequently, interpretation of interaction terms should be approached cautiously and validated in future studies with larger sample sizes. Moreover, this study was designed and conducted as a pilot study to explore the feasibility and potential effects of collagenase preconditioning combined with PRP and/or LED therapy on tendon healing in a small-animal in vivo model.

## 5. Conclusions and Recommendations

The findings of our study support the hypothesis of a potential conditioning effect of low concentrations of collagenase combined with LED and/or PRP treatments upon a partially incised tendon wound. Moreover, findings validate the in vivo partially tenotomized tendon model, supported by the histological findings (presented as metaplasia, disorganized matrix, and cell infiltrate). In addition, the established model conditioned with low concentrations of collagenase at the site of transection and treated with PRP and LED (λ = 630 nm and energy density = 4 J/cm^2^) for 21 days supports the potential of this treatment for improving the healing process, based on the histological findings. Future studies should explore time-dependent effects across all phases of tendon healing, include biomechanical assessments, and evaluate optimal dosing and delivery strategies for collagenase in combination with regenerative therapies to better translate findings into clinical application.

## Figures and Tables

**Table 1:** Study setup of animal groups.

Group Name (Number)	Collagenase Application	Partial Tenotomy of AT	4 J/cm^2^ LED	PRP Treatment
Normal Control (G1)	No	No	No	No
Model (G2 *)	No	Yes	No	No
Collagenase-conditioned (G3 *)	Yes	Yes	No	No
LED with a collagenase-conditioned (G4 **)	Yes	Yes	Yes	No
PRP with a collagenase-conditioned (G5 ***)	Yes	Yes	No	Yes
LED and PRP with a collagenase-conditioned (G6)	Yes	Yes	Yes	Yes
LED (G7 **)	No	Yes	Yes	No
PRP (G8 ***)	No	Yes	No	Yes
LED and PRP (G9)	No	Yes	Yes	Yes

* Rats received only placebo treatment (0.2 mL saline weekly and sham LED every other day). ** Rats received 0.2 mL saline weekly. *** Rats received sham LED every other day. AT: Achilles tendon.

**Table 2:** Features of the four-point scoring system for histological criteria.

Criterion	Four-Point Scoring			
	4	3	2	1
Collagen fibers	Normal wavy	Slight change	Moderate change	Disorganized
Cellularity	Normal	Slight increase	Moderate increase	High increase
Nucleus shape	Flattened	Semi-flattened	Semi-rounded	Rounded

**Table 3:** Summary of Partial Eta-Squared (partial η²) results.

Tested Variables	Effect	Partial η²	Effect Size Category
Weight Change *	0.089	Small	Weak
Hemoglobin (HB) **	0.106	Small	Weak
White Blood Cells (WBCs) **	0.236	Small	Weak
Packed Cell Volume (PCV) **	0.077	Small	Weak
Creatinine **	0.385	Medium	Moderate
Alanine Transaminase (ALT) **	0.510	Large	Moderate
Aspartate Transaminase (AST) **	0.431	Large	Moderate
Creatine Phosphokinase (CPK) **	0.854	Large	Strong
Collagen Organization (Rank) *	0.150	Small	Weak
Cellularity (Rank) *	0.127	Small	Weak
Nucleus Shape (Rank) *	0.166	Small	Weak
Overall Histology Score (Rank) *	0.107	Small	Weak

* Aligned Ranks Transformation Two-Way ANOVA. ** Standard Two-Way ANOVA.

**Table 4:** Summary of the R^2^ Effect sizes results.

Tested Variables	Effect	Partial η²	Effect Size Category
Weight Change *	Phototherapy	0.029	Small
	Collagenase	0.014	Small
	Interaction	0.053	Small
Hemoglobin (HB) **	Phototherapy	0.038	Small
	Collagenase	0.052	Small
	Interaction	0.029	Small
White Blood Cells (WBC) **	Phototherapy	0.198	Large
	Collagenase	0.005	Small
	Interaction	0.053	Small
Packed Cell Volume (PCV) **	Phototherapy	0.053	Small
	Collagenase	0.026	Small
	Interaction	0.004	Small
Creatinine **	Phototherapy	0.160	Large
	Collagenase	0.243	Large
	Interaction	0.123	Medium
Alanine Transaminase (ALT) **	Phototherapy	0.275	Large
	Collagenase	0.158	Large
	Interaction	0.184	Large
Aspartate Transaminase (AST) **	Phototherapy	0.135	Medium
	Collagenase	0.378	Large
	Interaction	0.040	Small
Creatine Phosphokinase (CPK) **	Phototherapy	0.641	Large
	Collagenase	0.304	Large
	Interaction	0.555	Large
Collagen Organization (Rank) *	Phototherapy	0.014	Small
	Collagenase	0.014	Small
	Interaction	0.129	Medium
Cellularity (Rank) *	Phototherapy	0.048	Small
	Collagenase	0.001	Small
	Interaction	0.065	Medium
Nucleus Shape (Rank) *	Phototherapy	0.012	Small
	Collagenase	0.012	Small
	Interaction	0.126	Medium
Overall Histology Score (Rank) *	Phototherapy	0.012	Small
	Collagenase	0.001	Small
	Interaction	0.086	Medium

* Aligned Ranks Transformation Two-Way ANOVA. ** Standard Two-Way ANOVA.

## Data Availability

The original contributions presented in this study are included in the article/Supplementary Material. Further inquiries can be directed to the corresponding author.

## Supplementary Materials

The following supporting information can be downloaded at https://www.mdpi.com/article/10.3390/biomedicines13051214/s1. File S1: Normality and homogeneity results supplementary Tables S1 and S2; File S2: Overall model and Independent Factors Two-Way ANOVA results; File S3: GraphPad Prism Data files and Figures.
