# Supplementary material for: Effects of Collagenase Preconditioning on Partially Incised Rat Tendon Treated with Light-Emitting Diodes and Platelet-Rich Plasma
Source: Biomedicines. 2025 May 16;13(5):1214. doi: 10.3390/biomedicines13051214 (PMC12109192; doi:10.3390/biomedicines13051214)
Supplement: Supplementary file 1 [file biomedicines-13-01214-s001.zip › File S1_Normality and Homogeneity Results Tables 1&2.pdf]

Table S1. Summary of the Normality Test Results.

Tested Variable	Shapiro-Wilk p-value	Normality
**Weight Change***	0.004	**No**
**HB**	0.508	Yes
**WBC**	0.344	Yes
**PCV**	0.370	Yes
**Creatinine**	0.864	Yes
**ALT**	0.792	Yes
**AST**	0.405	Yes
**CPK**	0.060	Yes
**Collagen Fiber Organization***	0.002	**No**
**Cellularity***	0.001	**No**
**Nucleus Shape***	0.002	**No**
**Overall Score***	0.001	**No**

***: Original and Transformed Data**

Table S2. Summary of the Homogeneity of Variances Test Results.

Tested Variable	Levene's Test p-value	Homogeneity
**Weight Change***	0.421	Yes
**HB**	0.318	Yes
**WBC**	0.232	Yes
**PCV**	0.171	Yes
**Creatinine**	0.359	Yes
**ALT**	0.276	Yes
**AST**	0.385	Yes
**CPK**	0.454	Yes
**Collagen Fiber Organization***	0.111	Yes
**Cellularity***	0.570	Yes
**Nucleus Shape***	0.281	Yes
**Overall Score***	0.283	Yes

***: Ranked Variables**
