# Supplementary material for: Effects of Collagenase Preconditioning on Partially Incised Rat Tendon Treated with Light-Emitting Diodes and Platelet-Rich Plasma
Source: Biomedicines. 2025 May 16;13(5):1214. doi: 10.3390/biomedicines13051214 (PMC12109192; doi:10.3390/biomedicines13051214)
Supplement: Supplementary file 1 [file biomedicines-13-01214-s001.zip › File S2_Overall model and Independant Factors Two-Way ANOVA results.pdf]

Table S3. Summary of the Two-Way ANOVA results for biochemical, hematological, and histological features.

Outcome	Significant Overall Model	Significant Factors
**Cellularity**	No ($p = 0.476$)	None
**Collagen Organization**	No ($p = 0.343$)	None
**Nucleus Shape**	No ($p = 0.267$)	None
**Overall Score**	No ($p = 0.603$)	None
**Weight Change**	No ($p = 0.884$)	None
**HB**	No ($p = 0.715$)	None
**WBC**	No ($p = 0.144$)	Phototherapy ($p = 0.037$)
**PCV**	No ($p = 0.860$)	None
**Creatinine**	No ($p = 0.106$)	Collagenase ($p = 0.014$)
**ALT**	**Yes ($p = 0.015$)**	None
**AST**	No ($p = 0.057$)	Collagenase ($p = 0.001$)
**CPK**	**Yes ($p < 0.001$)**	Phototherapy ($p < 0.001$), Collagenase ($p = 0.005$), Interaction ($p < 0.001$)
